# Predicting the Key Genes Involved in Aortic Valve Calcification Through Integrated Bioinformatics Analysis

**DOI:** 10.3389/fgene.2021.650213

**Published:** 2021-05-11

**Authors:** Dinghui Wang, Tianhua Xiong, Wenlong Yu, Bin Liu, Jing Wang, Kaihu Xiao, Qiang She

**Affiliations:** Department of Cardiology, The Second Affiliated Hospital of Chongqing Medical University, Chongqing, China

**Keywords:** CAVD, chemokines, immune cells, CXCL13, CCL19, CXCL8, macrophages, bioinformatics analysis

## Abstract

**Background:** Valvular heart disease is obtaining growing attention in the cardiovascular field and it is believed that calcific aortic valve disease (CAVD) is the most common valvular heart disease (VHD) in the world. CAVD does not have a fully effective treatment to delay its progression and the specific molecular mechanism of aortic valve calcification remains unclear.

**Materials and Methods:** We obtained the gene expression datasets GSE12644 and GSE51472 from the public comprehensive free database GEO. Then, a series of bioinformatics methods, such as GO and KEGG analysis, STING online tool, Cytoscape software, were used to identify differentially expressed genes in CAVD and healthy controls, construct a PPI network, and then identify key genes. In addition, immune infiltration analysis was used via CIBERSORT to observe the expression of various immune cells in CAVD.

**Results:** A total of 144 differential expression genes were identified in the CAVD samples in comparison with the control samples, including 49 up-regulated genes and 95 down-regulated genes. GO analysis of DEGs were most observably enriched in the immune response, signal transduction, inflammatory response, proteolysis, innate immune response, and apoptotic process. The KEGG analysis revealed that the enrichment of DEGs in CAVD were remarkably observed in the chemokine signaling pathway, cytokine-cytokine receptor interaction, and PI3K-Akt signaling pathway. Chemokines CXCL13, CCL19, CCL8, CXCL8, CXCL16, MMP9, CCL18, CXCL5, VCAM1, and PPBP were identified as the hub genes of CAVD. It was macrophages that accounted for the maximal proportion among these immune cells. The expression of macrophages M0, B cells memory, and Plasma cells were higher in the CAVD valves than in healthy valves, however, the expression of B cells naïve, NK cells activated, and macrophages M2 were lower.

**Conclusion:** We detected that chemokines CXCL13, CXCL8, CXCL16, and CXCL5, and CCL19, CCL8, and CCL18 are the most important markers of aortic valve disease. The regulatory macrophages M0, plasma cells, B cells memory, B cells naïve, NK cells activated, and macrophages M2 are probably related to the occurrence and the advancement of aortic valve stenosis. These identified chemokines and these immune cells may interact with a subtle adjustment relationship in the development of calcification in CAVD.

## Background

Valvular heart disease is obtaining growing attention in the cardiovascular field and it is believed that calcific aortic valve disease (CAVD) is the most common valvular heart disease (VHD) worldwide (Iung and Vahanian, 2011; Liu et al., 2017). It is also the predominant causation of aortic valve stenosis (Iung and Vahanian, 2011). CAVD presents with an escalating fibrous calcification remodeling and pathological incrassation of aortic valves, which evolves for a long time, inducing a serious block of cardiac outflow and ultimately reducing the mobility of leaflets (Lindman et al., 2016). Aortic stenosis (AS), the most common clinical manifestation of CAVD, its prevalence increases with age, impacting about 9% of patients older than 80 years. In addition, aortic sclerosis is obviously more widespread, impacting 25–30% of patients older than 65 years, and progresses to AS at a ratio of approximately 2% every year (Coffey et al., 2014; Otto and Prendergast, 2014). It is assumed that CAVD is becoming a major societal and economic burden for the aging world that is likely to be confirmed in the near future (Osnabrugge et al., 2013). In spite of the progress on radical therapies, such as valve replacement and intervention therapy already used for aortic stenosis, still, the specific molecular mechanism of aortic valve calcification remains unclear. Therefore, it is of great necessity to understand the evolution of CAVD and explore the key molecules for better treatment of aortic stenosis.

As is well-known, CAVD develops slowly and the severity of each stage is different. In the beginning, there is only mild valve thickening while at terminal stage, grievous calcification is accompanied by impaired leaflet movement, aortic valve stenosis and clinical symptoms such as heart pits, dyspnea, and angina (Freeman and Otto, 2005; Rajamannan et al., 2007). In the past, CAVD was deemed to be a retrograde procedure owing to the time-conditioned abrasion of valves and calcification deposition passively. At present, lots of convincing evidences have implied that CAVD was an unasked, multifaceted process, which is concerned with the sedimentation of chylomicrons, VLDL, LDL, active valve calcification, ossification of valvular interstitial cells, and so on (Freeman and Otto, 2005). More and more researches suggested that signal transduction channels referred to the progress of aortic calcification consisted of various growth factors, cytokines, and tumor necrosis factors (O’Brien et al., 2002; Jian et al., 2003; Kaden et al., 2005). Since statins and angiotensin-converting enzyme inhibitors failed to retard CAVD progression, there were no effective medical therapies (Lindman et al., 2013; Nishimura et al., 2014). When CAVD progresses to an advanced stage with terrible clinical manifestations, the most effective measures are surgery and transcatheter aortic valve replacement (TAVR), but the actual situation is that these procedures are related to higher costs, unavoidable risk of death, perioperative and long-run complications such as complications of lifelong anti-coagulation therapy and reoperation of artificial valve insufficiency (Lindman et al., 2016).

Hence, it is necessary for us to make a deeper discernment of the latent mechanisms of CAVD and mine certain key genes which perhaps are curative targets. In the recent years, the high-throughput sequencing information acquired via chip techniques helped us confirm the differentially expressed genes (DEGs) and their functions, excavate their pathways related to the advancement of various complex disorders (Zhao et al., 2018). Through comprehensive bioinformatics analysis of public genetic data, we could be able to conduct a secondary data digging and identify biomarkers related to diseases (Lei et al., 2019). Furthermore, we investigated the correlation between key molecules and immune cells to deeply and comprehensively comprehend the immune mechanism among the developing process in CAVD.

## Materials and Methods

### Affymetrix Microarray Data

The gene expression profiles with series numbers GSE12644 and GSE51472 were obtained from the Expression Omnibus Gene (GEO) database^1^ ^,2^ NCBI (Lei et al., 2019). These two datasets were genetic expression profiles of aortic valves from patients with calcified aortic valve diseases and healthy controls, which were detected on the GPL570 platform and founded on the Affymetrix Human Genome U133 Plus 2.0 array (Li et al., 2015). GSE12644 contains gene expression profile of 10 normal and 10 calcified stenotic human aortic valves from Canada. GSE51472 contains 15 human aortic valves from during the different stages of development of calcified aortic valve disease (normal, sclerotic, calcified) from Finland. All the participants were white male. In order to analyze it more conveniently, we removed the five sclerotic valve samples after merging and batch calibration from all these data. Moreover, we also obtained two data sets related to CAVD (GSE83453 and GSE26953) from GEO to further verify and analyze the results of immune infiltration. GSE83453 included 10 calcified aortic valves, nine stenotic aortic valves, and eight normal aortic valves, and GSE26953 contained 24 mRNAs of human aortic valve endothelial cells under the following conditions: laminar flow and shear stress.

### Data Preprocessing and Identification of DEGs

We obtained the original data of these two chips, GSE12644 and GSE51472, and then read them through the R package (version: 4.2.0). Subsequently, the two data sets were merged and batch corrected. In addition, we made use of the annotation configuration file offered by the platform to annotate probes and discard probes unmatched.(Zhang et al., 2019). Limma package in R was applied to screen the DEGs of CAVD with *P* value < 0.05, | log2 (Fold Change)| ≥ = 1. Finally, we distinguished 49 genes with an increased expression and 95 genes with a decreased expression in the combined matrix. By processing the heatmap package of R, we accomplished hierarchical clustering and visualization. As for GSE83453 and GSE26953, we just utilized the annotation configuration file to annotate probes.

### GO Enrichment and KEGG Analysis of DEGs

The powerful online bioinformatics database, Database for Annotation, Visualization, and Integrated Discovery (DAVID^3^), furnishes investigators with an all-round range of functional annotation tools to solve the biological implications of large gene lists (Yin et al., 2020). In this research, we used DAVID to aggregate the functions of DEGs including the screening of significant biological processes, molecular functions, cellular components, and as well as, identifying pathways related to these DEGs (Zou Z. B. et al., 2019). In order to obtain the visual results of the function and pathway terms, we used the R package clusterProfiler to obtain the histogram of the top six most important GO functions separately and the bubble chart of the signal pathways.

### Establishment of the Protein-Protein Interaction (PPI) Network

On account of the Search tool for the retrieval of interacting genes/proteins (STRING^4^), our PPI network among the DEGs-encoded proteins were established successfully (Zhao et al., 2018). Using the Network Analyzer available in the Cytoscape software (version: 3.7.2), the PPIs network was visualized. Using the Network Analyzer provided in the Cytoscape software (version: 3.7.2), you can see the PPI network. CytoHubba in Cytoscape is mainly used to rank nodes in the network through its network function, and provides 11 topological analysis methods (Ali et al., 2016). Of these 11 means, MCC can predict the essential proteins in the PPI network more accurately and finally, we obtained the 10 core genes of the PPI network according to the degree score.

### Immune Infiltration Analysis and Immune Score/Stromal Score

CIBERSORT^5^, a robust online tool, utilizes a deconvolution algorithm to analyze the relationship between gene expression and infiltrating immune cells (Ali et al., 2016). Combined and corrected micro array matrix was uploaded to the CIBERSORT algorithm running to deduce the percentages of 22 kinds of infiltrating immunocytes and then selected samples with *P* < 0.05 to the final study cohort (Deng et al., 2020). The analyzed immunocytes contained macrophages, dendritic cells (DC), B cells, CD4+ T cells, CD8+ T cells, neutrophils, and several naive cells. Subsequently, based on R, the results of the immune infiltration analysis are visualized in different ways like bar plot, heatmap, and co-heatmap. ESTIMATE algorithm calculates the immune score and stromal score from the matrix of GSE83453 and GSE26953 data sets; in this algorithm, the immune and stromal scores are calculated by analyzing the specific gene expression characteristics of immune and stromal cells to predict the infiltration of non-tumor cells.

### Correlation Analysis of Hub Gene and Lipid Metabolic Markers

In order to analyze the relationship between hub genes and lipid metabolism related biomarkers, firstly, we used the script in the Perl^6^ software to extract the identified hub genes and metabolism-related biomarkers. Subsequently, the limma package containing the Cor function of the R language was used to analyze the correlation coefficients and draw correlation pictures between the two variables (Tang et al., 2020). According to statistical standards, the correlation coefficient r greater than 0.4 is a positive correlation, less than −0.4 is a negative correlation, and it is considered that the *p* value that is less than 0.05 has a statistical difference (Wang et al., 2019).

## Results

### Identification of Differentially Expressed Genes in CAVD

**FIGURE 1:**
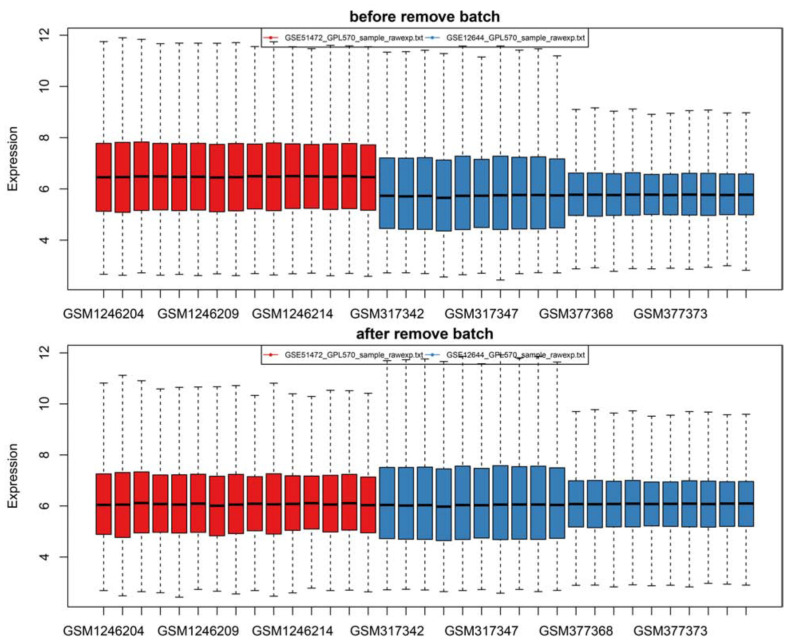
There are two batches of samples in the original data as revealed by the box plot and the expression profile was successfully standardized to a comparable level by pretreatment and batch-effects adjustment.

**FIGURE 2:**
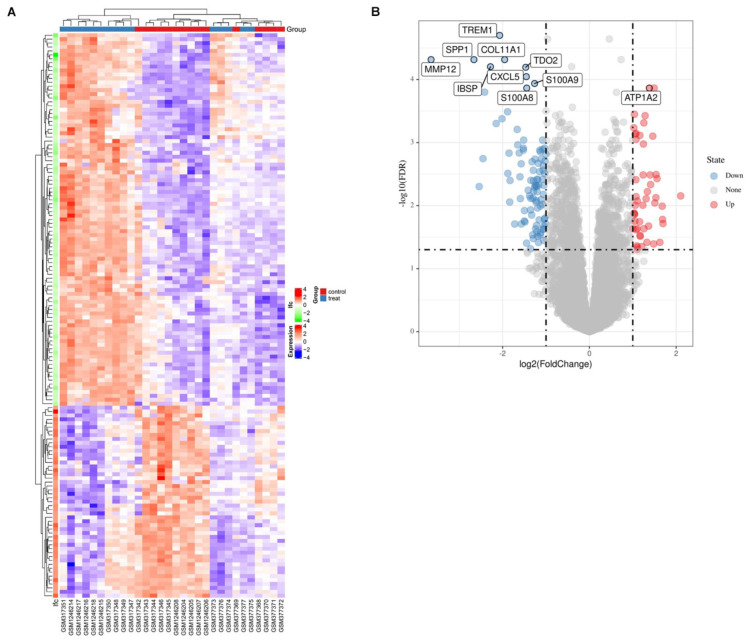
The identification of EDGs. **(A)** The heat-map of differential expression genes (*P* < 0.05, | logFC| > 1). Up-regulated genes were in red and down-regulated genes were in blue. **(B)** Volcano plot of genes detected in CAVD, red dots represent upregulated genes and blue dots represent downregulated genes.

Firstly, after merging the GSE12644 and GSE51472 datasets, we removed the batch differences between the two gene expression data sets (Figure 1). Subsequently, there are 144 DEGs altogether satisfying the criterion of | log2FC| ≥ = 1 and *P* value < 0.05, containing 49 genes with an increased expression and 95 genes with a decreased expression in CAVD compared to the healthy samples. Through both the heat map and volcano plot (Figures 2A,B), we can see the remarkable difference in the distribution between each data set.

### GO and KEGG Functional Analysis of DEGs in CAVD

**FIGURE 3:**
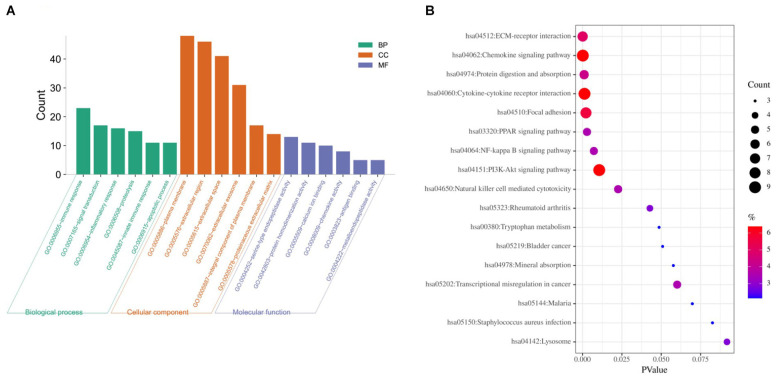
GO and KEGG pathway enrichment analysis of DEGs between CAVD and normal controls. **(A)** The top six GO analysis results of DEGs. **(B)** Pathway analyses results of DEGs (top 20 according to adjusted *P* value). GO, gene ontology; KEGG, Kyoto Encyclopedia of Genes and Genomes.

Based on DAVID, we executed the GO and KEGG functional annotation analysis for investigating the specific biological function classification of DEGs globally (Zou R. et al., 2019). GO function annotation results displayed that changes at biological process (BP) were observably focused in immune response, signal transduction, inflammatory response, proteolysis, innate immune response, and apoptotic process (Figure 3A). The most enriched molecular function (MF) annotations were serine-type endopeptidase activity, protein homodimerization activity, calcium ion binding, chemokine activity, antigen binding, and metallo endopeptidase activity. Changes of DEGs significantly in cell component (CC) were mostly in the plasma membrane, extracellular region, extracellular space, extracellular exosome, integral component of plasma membrane, and proteinaceous extracellular matrix. In addition, the results of the KEGG pathway analysis in bubble chart revealed that DEGs were remarkably concentrated in the chemokine signaling pathway, cytokine-cytokine receptor interaction, PI3K-Akt signaling pathway, focal adhesion, ECM-receptor interaction, and protein digestion and absorption (Figure 3B). Some more details of the GO and KEGG analyses can be seen in Tables 1, 2.

**TABLE 1:** The GO functional enrichment analyses of DEGs ranked by count.

Term	Count	Genes	*P*-value
**GO-BPs**			
GO:0006955∼immune response	23	ADAMDEC1, CXCL8, GZMA, GPR65, AQP9, C5AR1, PRG4, PPBP, CXCL13, CXCL5, JCHAIN, FCGR3A, FCGR3B, CCL8, IGKC, TRBC1, CTSG, IGLC1, PKHD1L1, CCL19, IL7R, CCL18, and FCGR1B	1.08E-12
GO:0007165∼signal transduction	17	CXCL8, TNFAIP6, C5AR1, TRHDE, RASGRP1, CXCL5, ADRA2A, GREM1, CCL8, PLAU, WIF1, KIT, CLEC5A, NDP, IL7R, CCL18, and S100A9	0.016322305
GO:0006954∼inflammatory response	16	CXCL8, TNFAIP6, C5AR1, LY86, PPBP, CXCL13, CXCL5, CCL8, KIT, SPP1, CHI3L1, CCL19, CCL18, S100A9, SCG2, and S100A8	2.20E-07
GO:0006508∼proteolysis	15	FCN1, CPA3, ADAMDEC1, TPSB2, MMP1, TRHDE, MMP9, MMP12, GZMK, MMP13, RELN, IGKC, PLAU, CTSG, and IGLC1	3.05E-05
GO:0045087∼innate immune response	11	IGHM, C6, FCER1G, IGKC, CLEC5A, LY86, IGLC1, TREM1, S100A9, S100A8, and JCHAIN	0.001788216
GO:0006915∼apoptotic process	11	CD2, SHC4, GREM1, GZMA, GPR65, C5AR1, LY86, GZMB, CHI3L1, S100A9, and S100A8	0.012113741
**GO-CCs**			
GO:0005886∼plasma membrane	48	SHC4, GPM6A, IGHM, GPR65, C5AR1, AQP9, C2ORF88, GPR83, ATP1A2, GPR84, IFI30, TREM1, RASGRP1, CXCL16, FCGR3A, RELN, FCGR3B, IGKC, PRKAR2B, PLAU, LAMP3, TRBC1, CLEC5A, HMOX1, SCN7A, IGLC1, CTSG, FCGR1B, PRIMA1, FCER1G, VCAM1, CD93, ANXA3, LY86, SLC16A10, ADRA2A, VMP1, CD2, VNN2, KIT, GPR160, PCDHB5, PLPP3, IL7R, STEAP1, S100A9, S100A8, and CLEC4G	8.05E-04
GO:0005576∼extracellular region	46	FCN1, ADAMDEC1, TPSB2, CXCL8, COL11A1, TNC, NDNF, CXCL13, IFI30, THBS2, TREM1, PLA2G7, CHRDL1, CXCL5, CXCL16, JCHAIN, APELA, C6, IBSP, IGKC, PLAU, WIF1, SPP1, COL10A1, IGLC1, CTSG, CCL19, PLTP, CPA3, CHST9, MMP1, GZMA, ADIPOQ, PRG4, PPBP, MMP9, MFAP5, CD2, MMP12, GZMK, IGSF10, MMP13, COL6A6, IL7R, S100A9, and S100A8	1.19E-15
GO:0005615∼extracellular space	41	IGHM, CXCL8, TNFAIP6, TNC, CXCL13, CHRDL2, CXCL5, CXCL16, JCHAIN, APELA, RELN, CCL8, IBSP, IGKC, PLAU, SPP1, HMOX1, IGLC1, CTSG, NDP, CCL19, CCL18, PLTP, TIMP4, CTHRC1, CPA3, VCAM1, MYOC, ADIPOQ, LY86, PPBP, MMP9, GREM1, MMP13, KIT, CHI3L1, PKHD1L1, S100A9, SCG2, S100A8, and ANGPTL1	1.04E-14
GO:0070062∼extracellular exosome	31	GPM6A, IGHM, ITLN1, TRHDE, COCH, JCHAIN, FCGR3A, C6, FCGR3B, ALDH2, IGKC, TSPAN8, PRKAR2B, PLAU, SPP1, ACP5, IGLC1, CTSG, VCAM1, MYOC, ANXA3, ADIPOQ, MMP9, KRT18, KRT14, CHI3L1, MNDA, PLPP3, S100A9, S100A8, and ANGPTL1	0.022687598
GO:0005887∼integral component of plasma membrane	17	CD52, FCER1G, SCARA5, GPR65, AQP9, C5AR1, LAPTM5, GPR83, SLC16A10, GPR84, TRHDE, ADRA2A, CD2, TSPAN8, CLEC5A, PLPP3, and STEAP1	0.059028895
GO:0005578∼proteinaceous extracellular matrix	14	MYOC, MMP1, COL11A1, NDNF, MMP9, COCH, MMP12, MMP13, RELN, COL10A1, CHI3L1, COL6A6, TIMP4, and CTHRC1	9.98E-08
**GO-MFs**			
GO:0004252∼serine-type endopeptidase activity	13	FCN1, TPSB2, MMP1, GZMA, GZMB, MMP9, MMP12, GZMK, MMP13, IGKC, PLAU, CTSG, and IGLC1	3.25E-07
GO:0042803∼protein homodimerization activity	11	CD2, BCL2A1, GZMA, ID1, KIT, ADIPOQ, HMOX1, NDP, RASGRP1, ADRA2A, and JCHAIN	0.038761584
GO:0005509∼calcium ion binding	10	MMP12, MMP13, CD93, ANXA3, MMP1, PCDHB5, THBS2, S100A9, RASGRP1, and S100A8	0.075449087
GO:0008009∼chemokine activity	8	CCL8, CXCL8, PPBP, CCL19, CXCL13, CCL18, CXCL5, and CXCL16	5.78E-08
GO:0003823∼antigen binding	5	IGHM, IGKC, IGLC1, IL7R, and JCHAIN	0.006624345
GO:0004222∼metalloendopeptidase activity	5	MMP12, ADAMDEC1, MMP13, MMP1, and MMP9	0.009131648

**TABLE 2:** The KEGG pathway analysis of DEGs ranked by count.

Term	Count	Genes	*P*-value
KEGG_PATHWAY			
hsa04062:Chemokine signaling pathway	9	SHC4, CCL8, CXCL8, PPBP, CCL19, CXCL13, CCL18, CXCL5, and CXCL16	2.12E-04
hsa04060:Cytokine-cytokine receptor interaction	9	CCL8, CXCL8, PPBP, CCL19, CXCL13, IL7R, CCL18, CXCL5, and CXCL16	0.001258766
hsa04151:PI3K-Akt signaling pathway	9	RELN, IBSP, COL11A1, KIT, SPP1, TNC, COL6A6, THBS2, and IL7R	0.010615579
hsa04510:Focal adhesion	8	SHC4, RELN, IBSP, COL11A1, SPP1, TNC, COL6A6, and THBS2	0.002157992
hsa04512:ECM-receptor interaction	7	RELN, IBSP, COL11A1, SPP1, TNC, COL6A6, and THBS2	1.09E-04
hsa04974:Protein digestion and absorption	6	CPA3, COL11A1, COL10A1, COL6A6, ATP1A2, and SLC16A10	0.001034968
hsa03320:PPAR signaling pathway	5	ACADL, MMP1, ADIPOQ, PLIN1, and PLTP	0.002827573
hsa04064:NF-kappa B signaling pathway	5	VCAM1, CXCL8, BCL2A1, PLAU, and CCL19	0.007200464
hsa04650:Natural killer cell mediated cytotoxicity	5	SHC4, FCGR3A, FCGR3B, FCER1G, and GZMB	0.022607194
hsa05323:Rheumatoid arthritis	4	CXCL8, MMP1, ACP5, and CXCL5	0.042784539
hsa00380:Tryptophan metabolism	3	TDO2, ALDH2, and MAOA	0.048643268

### Establishment of PPI Network and Module Analysis

**FIGURE 4:**
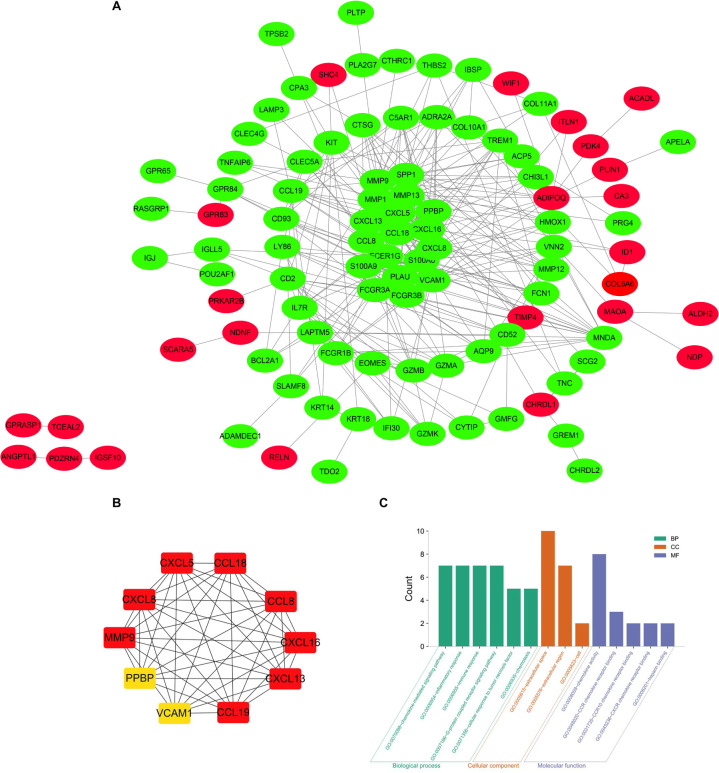
**(A)** The PPI network of DEGs identified from the two microarray datasets was constructed using Cytoscape, including 49 up-regulated genes and 95 down-regulated genes. The upregulated genes are marked in red and downregulated genes are marked in green. **(B)** The top 10 most significant genes were obtained from PPI network. DEG, differentially expressed gene; PPI protein, protein interaction. **(C)** The top 10 most significantly enriched GO terms of DEGs.

The precise PPI network was constructed based on the online tool STRING and then, was adjusted and visualized via the Cytoscape software (Cai et al., 2020). Ultimately, there were 344 edges and 102 nodes among this PPI network. The red dots represent the up-regulated genes and the green dots represent the down-regulated genes (Figure 4A). CytoHubba, a plug-in of the Cytoscape, was utilized to filter the most important genes and modules from the network. Top 10 genes (CXCL13, CCL19, CCL8, CXCL8, CXCL16, MMP9, CCL18, CXCL5, VCAM1, and PPBP) ranked by degree were selected (Figure 4B) as core molecules, the names, abbreviations, and functions for which are displayed in Table 3. In addition, BPs analysis showed that DEGs were related to the chemokine-mediated signaling pathway, inflammatory response, immune response, G-protein coupled receptor signaling pathway, cellular response to tumor necrosis factor, and chemotaxis, while CCs analysis showed that DEGs were chiefly concentrated on the extracellular space, extracellular region, and cell. As for MFs, the DEGs were mainly enriched in the chemokine activity, CCR chemokine receptor binding, CCR10 chemokine receptor binding, CXCR chemokine receptor binding, and heparin binding (Figure 4C).

**TABLE 3:** Summary of the function of 10 hub genes.

Top 10 in network string_interactions.tsv_MCC_top10 ranked by MCC method		
Gene symbol	Full name	Function
CXCL13	C-X-C motif chemokine ligand 13	B lymphocyte chemoattractant, independently cloned and named Angie, is an antimicrobial peptide and CXC chemokine strongly expressed in the follicles of the spleen, lymph nodes, and Peyer’s patches. It preferentially promotes the migration of B lymphocytes (compared to T cells and macrophages), apparently by stimulating calcium influx into, and chemotaxis of, cells expressing Burkitt’s lymphoma receptor 1 (BLR-1). It may therefore function in the homing of B lymphocytes to follicles
CCL19	C-C motif chemokine ligand 19	One of several CC cytokine genes clustered on the p-arm of chromosome 9. Cytokines are a family of secreted proteins involved in immunoregulatory and inflammatory processes. The CC cytokines are proteins characterized by two adjacent cysteines. The cytokine encoded by this gene may play a role in normal lymphocyte recirculation and homing. It also plays an important role in trafficking of T cells in thymus, and in T cell and B cell migration to secondary lymphoid organs. It specifically binds to chemokine receptor CCR
CCL8	C-C motif chemokine ligand 8	One of several chemokine genes clustered on the q-arm of chromosome 17. Chemokines form a superfamily of secreted proteins involved in immunoregulatory and inflammatory processes. The superfamily is divided into four subfamilies based on the arrangement of N-terminal cysteine residues of the mature peptide. This chemokine is a member of the CC subfamily which is characterized by two adjacent cysteine residues. This cytokine displays chemotactic activity for monocytes, lymphocytes, basophils, and eosinophils
CXCL8	C-X-C motif chemokine ligand 8	A member of the CXC chemokine family and is a major mediator of the inflammatory response. The encoded protein is commonly referred to as interleukin-8 (IL-8). IL-8 is secreted by mononuclear macrophages, neutrophils, and eosinophils, T lymphocytes, epithelial cells, and fibroblasts. It functions as a chemotactic factor by guiding the neutrophils to the site of infection. Bacterial and viral products rapidly induce IL-8 expression. IL-8 also participates with other cytokines in the proinflammatory signaling cascade and plays a role in systemic inflammatory response syndrome (SIRS). This gene is believed to play a role in the pathogenesis of the lower respiratory tract infection bronchiolitis, a common respiratory tract disease caused by the respiratory syncytial virus (RSV). The overproduction of this proinflammatory protein is thought to cause the lung inflammation associated with csytic fibrosis. This proinflammatory protein is also suspected of playing a role in coronary artery disease and endothelial dysfunction. This protein is also secreted by tumor cells and promotes tumor migration, invasion, angiogenesis and metastasis.
CXCL16	C-X-C motif chemokine ligand 16	CXCL16 (C-X-C Motif Chemokine Ligand 16) is a Protein Coding gene. Diseases associated with CXCL16 include Xanthogranulomatous Cholecystitis and Systemic Lupus Erythematosus. Among its related pathways are Signaling by GPCR and PEDF Induced Signaling. Gene Ontology (GO) annotations related to this gene include chemokine activity and low-density lipoprotein particle receptor activity.
MMP9	Matrix metallopeptidase 9	The matrix metalloproteinase (MMP) family are involved in the breakdown of extracellular matrix in normal physiological processes, such as embryonic development, reproduction, and tissue remodeling, as well as in disease processes, such as arthritis and metastasis. Most MMP’s are secreted as inactive proproteins which are activated when cleaved by extracellular proteinases. The enzyme encoded by this gene degrades type IV and V collagens. Studies in rhesus monkeys suggest that the enzyme is involved in IL-8-induced mobilization of hematopoietic progenitor cells from bone marrow, and murine studies suggest a role in tumor-associated tissue remodeling
CCL18	C-C motif chemokine ligand 18	One of several Cys-Cys (CC) cytokine genes clustered on the q arm of chromosome 17. Cytokines are a family of secreted proteins involved in immunoregulatory and inflammatory processes. The CC cytokines are proteins characterized by two adjacent cysteines. The cytokine encoded by this gene displays chemotactic activity for naive T cells, CD4+ and CD8+ T cells and nonactivated lymphocytes, but not for monocytes or granulocytes. This chemokine attracts naive T lymphocytes toward dendritic cells and activated macrophages in lymph nodes. It may play a role in both humoral and cell-mediated immunity responses
CXCL5	C-X-C motif chemokine ligand 5	This gene encodes a protein that is a member of the CXC subfamily of chemokines. Chemokines, which recruit and activate leukocytes, are classified by function (inflammatory or homeostatic) or by structure. This protein is proposed to bind the G-protein coupled receptor chemokine (C-X-C motif) receptor 2 to recruit neutrophils, to promote angiogenesis and to remodel connective tissues. This protein is thought to play a role in cancer cell proliferation, migration, and invasion
VCAM1	Vascular cell adhesion molecule 1	A member of the Ig superfamily and encodes a cell surface sialoglycoprotein expressed by cytokine-activated endothelium. This type I membrane protein mediates leukocyte-endothelial cell adhesion and signal transduction, and may play a role in the development of artherosclerosis and rheumatoid arthritis. Three alternatively spliced transcripts encoding different isoforms have been described for this gene.
PPBP	Pro-platelet basic protein	The protein encoded by this gene is a platelet-derived growth factor that belongs to the CXC chemokine family. This growth factor is a potent chemoattractant and activator of neutrophils. It has been shown to stimulate various cellular processes including DNA synthesis, mitosis, glycolysis, intracellular cAMP accumulation, prostaglandin E2 secretion, and synthesis of hyaluronic acid and sulfated glycosaminoglycan. It also stimulates the formation and secretion of plasminogen activator by synovial cells. The protein also is an antimicrobial protein with bactericidal and antifungal activity

### Immune Cell Infiltration of CAVD

**FIGURE 5:**
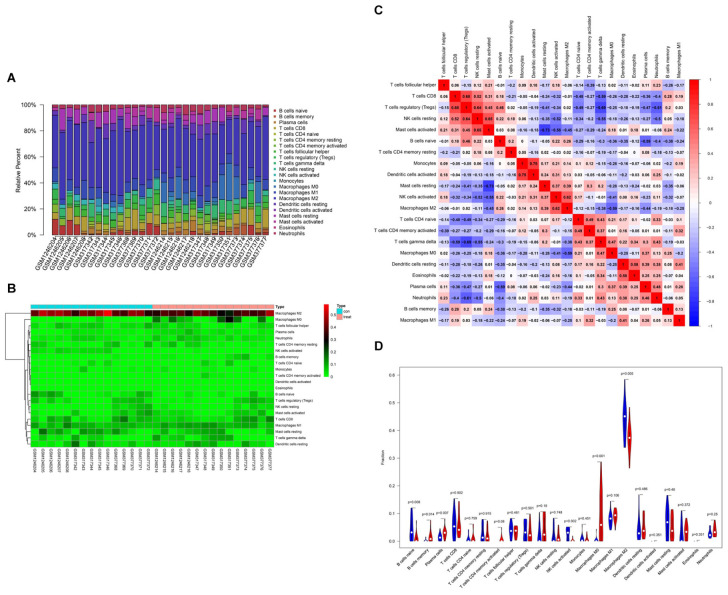
Summary of inferred immune cell subsets between CAVD and normal controls. **(A)** The relative percentage of 22 subpopulations of immune cells in 30 samples from GSE13985and GSE6054 datasets. **(B)** The heat-map of the 22 subpopulations of immune cells. **(C)** Correlation analysis based on 22 immune cell subpopulations. **(D)** The difference of immune infiltration between VACD and normal controls. (The normal controls group was marked as blue color and CAVD group was marked as red color. *P* values < 0.05 were considered as statistically significant).

**FIGURE 6:**
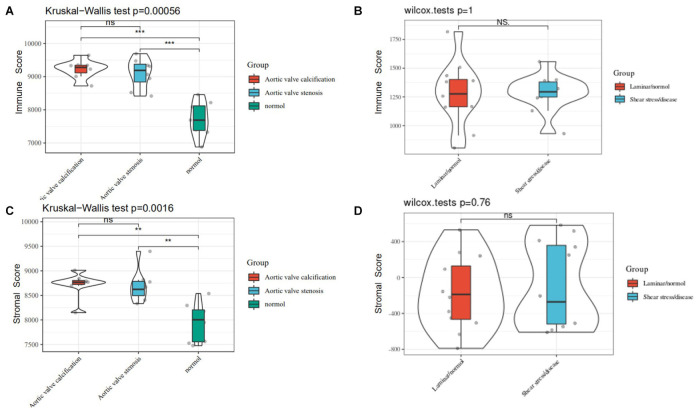
**(A)** The immune score of the normal, calcification, and stenosis groups of GSE83453. **(B)** The immune scores of the laminar flow group and the shear stress group in GSE26953. **(C)** The stromal scores of the normal, calcification, and stenosis groups. **(D)** The stromal scores of the laminar flow group and the shear stress group.

Based on CIBERSORT^7^, distinctions of 22 subpopulations of infiltration immune cells between the CAVD and normal valve tissues were identified (Zhao et al., 2020). Figures 5A,B could demonstrate the proportion of 22 kinds of immune cells vividly from 15 normal controls and 15 CAVD patients. In addition, we discovered that it was macrophages that accounted for the maximal proportion among these immune cells. Supplementary Table 1 showed the results of the immune cells infiltration specifically. Results of the correlation analysis between immune cells found that NK cells resting and mast cells activated had the most intense positive relationship with *r* = −0.65, yet mast cells activated and mast cells resting had the most obvious negative correlation with *r* = 0.73 (Figure 5C). The result of the analysis of the change in the trend of various immune cells showed that the infiltration of macrophages M0, B cells memory, and plasma cells in CAVD patients was higher compared with the healthy controls, but the infiltration of B cells naïve, NK cells activated, and macrophages M2 was lower (Figure 5D; *p* < 0.05). As shown in Figure 6A, the immune score of the normal aortic valve group was significantly lower than that of the calcification group and the stenosis group, but the immune scores of the laminar flow group and the shear stress group were not very different (Figure 6B). Similarly, the stromal scores of the normal group were significantly lower than that of the calcification and stenosis group (Figure 6C), but there was still no significant difference between the laminar flow group and the shear stress group (Figure 6D).

### Correlation Analysis of Metabolism Associated Genes

**FIGURE 7:**
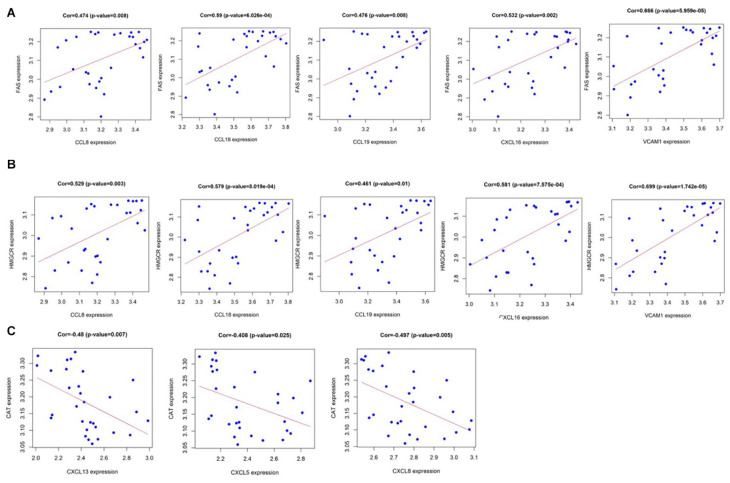
**(A)** CCL8, CCL18, CCL19, CXCL16, and VCAM1 are positively correlated with fatty acid synthase (FAS); and **(B)** CCL8, CCL18, CCL19, CXCL16, and VCAM1 are positively correlated with hydroxymethylglutaryl coenzyme A reductase (HMGCR). **(C)** CXCL13, CXCL5, and CXCL8 have a strong negative correlation with lecithin cholesterol acyltransferase (CAT). *P* value < 0.05.

Pearson correlation analysis shows that CCL8, CCL18, CCL19, CXCL16, and VCAM1 are positively correlated with fatty acid synthase (FAS) gene and hydroxymethylglutaryl coenzyme A reductase (HMGCR) gene among the 10 hub genes (Figures 7A,B), yet CXCL13, CXCL5, and CXCL8 have a strong negative correlation with the lecithin cholesterol acyltransferase (CAT) gene (Figure 7C). As we all know, the calcification process of CAVD involves lipid infiltration, FAS is a key factor in fat synthesis and HMGCR is a key enzyme that catalyzes the *de novo* synthesis of cholesterol in the body while CAT participates in reverse cholesterol transport (Zechner et al., 2012). This result further illustrates the close relationship between chemokines, lipid metabolism, and CAVD. More information on the 10 hub genes and metabolism-associated genes are shown in Supplementary Material.

## Discussion

Calcific aortic valve disease stands for a disease that covers the process from aortic valve sclerosis to aortic valve stenosis (Freeman and Otto, 2005). Incremental proofs verified that CAVD is an irreversible valvular lesion that remains irrecoverable though tremendous improvement in the surgical treatment strategy (Pawade et al., 2015). CAVD was primitively regarded as a retrograde procedure due to the gradual calcium sedimentation in aortic valve while getting older. It is generally accepted that CAVD is a complicated and active process and various physiological and pathological conditions are involved in the regulation like lipoprotein deposition, inflammatory response, activating RAAS, transformation of ECM, and cellular ossification (Mathieu and Boulanger, 2014; Kostyunin et al., 2019). Previous researches showed that valvular disease was widespread in most industrialized countries, where a decrement in the prevalence of rheumatic heart disease (RHD) has been associated with an enhancement in the popularity of degenerative etiologies (Coffey et al., 2014). Some studies have shown that valvular disease and coronary disease have some common risk factors, including hypertension, diabetes mellitus, dyslipidemia, cigarette, and so on (Nishimura et al., 2014). The pathophysiological process of CAVD principally devastates endothelial valve cells and valvular interstitial cells, exacerbating valvular calcification, making for stenosis of valve orifice, and blocking the left ventricular outflow tract (Dutta and Lincoln, 2018). The loading of valvular disease is anticipated to ascend, in consideration of the prolonged life expectancy and the current insufficiency of effective preventive measures, emphasizing the need to deepen our discernment of the pathophysiology of retrogressive heart valve diseases (Gould et al., 2013; Lindman et al., 2013). In recent decades, gene sequencing and bioinformatics technologies have developed dramatically, so it became possible to further analyze and utilize plentiful sequencing data (Zhao et al., 2020). In the present study, we screened significant DEGs between the aortic stenosis of CAVD and control samples from the GEO database. Furthermore, quite a lot of bioinformatics analysis was performed. The GO function annotation demonstrated that those DEGs were enriched in some BPs such as immune response, signal transduction, inflammatory response, proteolysis, innate immune response, and apoptotic process. KEGG pathway enrichment analysis uncovered that DEGs markedly related to chemokine signaling pathway, cytokine-cytokine receptor interaction, and PI3K-Akt signaling pathway. To explore molecular mechanism of CAVD, ten hub genes (CXCL13, CCL19, CCL8, CXCL8, CXCL16, MMP9, CCL18, CXCL5, VCAM1, and PPBP) connected with CAVD occurrence were explored out based on the STRING and Cytoscape software. We speculate that these important DEGs and their potential functions bring about the progression of CAVD in theory.

Among these hub genes, two kinds of chemokine ligands, CXCLs and CCLs, are very conspicuous. As we all know, the chemokine ligands family contains chemotactic cytokines or ligands and they are significant ingredients of the cross-communication among the assorted cell types in the inflammation or tumor microenvironment (Lacalle et al., 2017). Chemokine ligands mediate their activity by way of interacting with G protein coupled receptors that have unique 7 transmembrane architecture, hence becoming chemokine ligand/receptor pairs (Hussain et al., 2019). Chemokine ligand/receptor interactions include huge signal plasticity and complexity, which are crucial for fine-tuning chemotaxis of specific leukocyte subsets (Borroni et al., 2010). Firstly, the C-X-C motif chemokine ligands CXCL13, CXCL8, CXCL16, and CXCL5 are insiders of the CXC chemokine family. The protein encoded by CXCL13 is a chemokine ligand that plays a role in the homing of B lymphocytes to lymphoid follicles. Activated germinal center helper T cells are expressed at a high level. Cells with an up-regulated expression of CXCL13 receptor CXCL5 can induce chemotaxis. It induces B cells to enter follicles, and induces memory T cells in the paracortical area to return to follicles. Anne et al. reported a crucial function of CXCL13 and its receptor CXCR5 in durability and maintenance of cardiac structure in the case of pressure overload, possibly via modulating small leucine-rich proteoglycans (SLRPs) in ECM (Waehre et al., 2011). CXCL8, also known as interleukin-8, is a cytokine secreted by macrophages and epithelial cells and has a chemotactic effect on neutrophils to achieve its regulation of inflammation. It was reported that this proinflammatory protein may act on coronary artery disease even endothelial dysfunction and CXCL8 classically associates with an effective angiogenic role. The combination of CXCL8 with its receptors (IL-8RB/CXCR2) raises vascular permeability, and the increase of IL-8 level is positively correlated with the severity of prognosis of many diseases. Observations from Kokje et al. (2018) recognized that the high expression of CXCL8 is a clear distinguishing feature among abdominal aortic aneurysm disease (AAA) and atherosclerosis, and proved that there is a transcription mechanism demanding CXCL8 signal transduction in AAA. CXCL16 (C-X-C motif chemokine ligand 16) protein exists in two forms, transmembrane-bound type and soluble secretion type. The two forms have different biological effects and are related to each other. The transmembrane-bound type mainly functions as a scavenger receptor, and the soluble secreted type mainly functions as a chemokine. CXCL16 explicitly combines with Ox LDL, indicating that it probably relates to atherosclerosis (Shimaoka et al., 2000). There was indeed research deeming CXCL16 can have athero-protective effect but might make plaque unstable, yet CXCR6 can prevent myocardial ischemia (Izquierdo et al., 2014). Furthermore, the CXCL16 also induces a strong chemotactic response and calcium mobilization that may have a potential effect on the progression of CAVD. CXCL5 bound to G-protein coupled receptor and transmits cell signals recruiting neutrophils promoting angiogenesis and rebuilding connective tissue. In addition, CXCL5/ENA-78 is a pro-inflammatory chemokine pair that mediates CXCR2-dependent neutrophil transportation in humans (Layhadi et al., 2018). Researchers believed that platelets are the major source of CXCL5 in homeostasis, nevertheless, CXCL5 is mainly generated by lung epithelial cells in fierce infection (Koltsova and Ley, 2010). Performer studies showed that CXCL5 induces EMT to promote CRC cell migration via activating ERK/Elk-1/Snail, and enhances infiltration by activating AKT/GSK3β, thereby inhibiting β-catenin degradation (Zhao et al., 2017). Interestingly, Ravi et al. (2017) found that the plasma CXCL5 level is negatively correlated with CHD severity from clinical evidence.

Besides, the CC cytokines CCL19, CCL8, and CCL18 screened in the network are proteins containing two neighboring cysteine. CCL19 can function in lymphocyte recirculation and homing and is also a significant regulatory factor, which induces T cell activation, immune tolerance, and inflammatory reaction in the course of sequential immune monitoring and homeostasis. It reported that CCL19 and the paired CCR7 were are associated with autoimmune diseases like rheumatoid arthritis and enteropathy (Burman et al., 2005). Damås et al. (2007) found that CCL19 was expressed in the vascular plaques of ApoE -/- mice with atherosclerosis, homo’s carotid plaque and plasma of sufferers with CAD. Moreover, they also testified the plasma level of CCL19 clearly increased in cases with unstable angina pectoris than the sick with stable angina pectoris and healthy control patients (Damås et al., 2007). CCL8 is another member of the CC subfamily, which CCL8 has chemotactic activity on mononuclear cells, leukomonocytes, basophilic granulocytes, and eosinophilic granulocytes. Through enlisting white blood cells to inflammatory places, CCL8 contributes to tumor-interrelated neutrophils infiltration and antivirotic condition in opposition to HIV (Lacalle et al., 2017). The cytokine CCL18 exerts obvious chemoattractant activities on immature T cells, CD4+ and CD8+ T cells, and resting lymphocytes. It recruits immature T lymphocytes to DCs and actuated macrophages in lymph glands and functions in humoral and cellular immune processes (Murphy et al., 2000). Matrix metalloproteinase (MMP) are related to the decomposition of ECM in natural physiological events like fetal development, engendering, and tissue reconstitution, along with in clinical courses like arthrophlogosis and metastatic tumor (Kluger et al., 2013). The function of MMP9 is to debase collagen IV and V. Researches on macaques have shown that MMP9 is concerned with IL-8-elicited mobilization of bone marrow-derived blood ancestral cells and additionally, some studies indicated that MMP9 acted on tumor-related tissue reconstitution (Pruijt et al., 1999). Vascular cell adhesion molecule 1 (VCAM1), belongs to the Ig superfamily, encodes sialoglycoprotein on endothelial cell surface activated by cytokine. VCAM1 is a type I membrane protein as well mediating the adhesion of leukocytes to endothelial cell and signaling transduction and functioning in the development of atherosclerosis and rheumatoid arthritis (Dessein et al., 2005). It is reported that VCAM1/ITGA4/ITGB1 interactivity possibly worked on the pathophysiological process of immunological reactions and leukocyte traffic to inflammatory spots. Pro-platelet basic protein (PPBP), alternately named as CXCL7, is set free abundantly from activated platelets and participates in vascular injury responses (Stankiewicz et al., 2014). PPBP is also an effective chemotactic agent and neutrophil activator. What is more, researched suggested that it stimulated a variety of cellular physiological courses containing DNA synthesis, mitosis, anaerobic glycolysis, cytosolic cAMP amassing, PG-E2 release, and so on (Brown et al., 2017). In a nutshell, it seems that the inflammatory response is very active during the calcification of CAVD.

To search immunocytes infiltration in the microenvironment of CAVD, CIBERSORT was employed to manage a thorough estimation of the data obtained from GEO previously. A growing infiltration of macrophages M0, B cells memory, plasma cells and a dropping infiltration of B cells naïve, NK cells activated, macrophages M2 were identified that may be connected with the incidence and progression of CAVD. Former researchers have demonstrated the existence of inflammatory T cells which accounted for atherosclerotic lesions forming at an early stage of aortic valve disease and the presence of C-reactive protein in valve samples of candidates suffering from aortic stenosis after percutaneous aortic valve replacement illustrating an inflammatory state during calcification (Galante et al., 2001; Rajamannan et al., 2005). Notably, we found that the macrophage0 population were significantly high expression in the calcified valve. This is consistent with previous research results (Cote et al., 2013). In another study, aortic valve interstitial cells (AVICs), the main components of valves, were treated with conditioned media from M1 macrophages. The results displayed that expression of osteogenic calcification markers increased at transcription and translation levels, antibody barricade the inflammatory factors TNF and/or IL-6 decreased the calcification *ex vivo* indicating a pro-calcific effect of macrophages (Li et al., 2017). Research showed that chronic inflammation with infiltration of lymphocytes, phagocytes, tissue cells, and mastocytes existed in calcific aortic stenosis (Steiner et al., 2012). In addition, it was reported that NK cells gathered in the valve and circulation of patients with CAVD, which is related to the addition of valve pressure gradient (Mazur et al., 2018). From another perspective, correlation analysis in our study showed that the identified key genes CCL8, CCL18, CCL19, CXCL16, and VCAM1 were related to lipid metabolism biomarkers, which were closely related to the occurrence of CAVD from a biochemical point of view. A study on senile calcific aortic valve disease showed that plasma lipoprotein(a) was an independent risk factor for CAVD (Zhanjing et al., 2019). It can be speculated that cytokines and lipid metabolites may also have lipid-cytokine-chemical cascades that is similar to neutrophil-driven joint inflammation in immune complex-mediated arthritis and eosinophilic pneumonia driven by TH2 cells (Sadik and Luster, 2012). Additionally, experiments have shown that the expression of chemokines can directly affect the production of cytokines and cellular immune responses (Murphy et al., 2000). From the results of our study, we reasonably believe that chemokines and immune cells interact with each other during inflammation and even calcification of CAVD. For example, CXCL13 obviously leads to the recruitment of B cells and the production of LT-a β. In turn, LT-a β can stimulate the secretion of CXCL13 (Lalani et al., 1999). In addition, many chemokines secreted by lymphocytes have their own chemotactic effects. The concentration of lymphocytes in the microenvironment can be significantly expanded. Chemokines are also expressed on the surface of immune cells such as T or B cells. The expression of CCR7 causes dendritic cells to accumulate in the lymph. The fluid is infused into the human tube and T cells are distributed in the lymph nodes. Both CCL21 and CCL19 can bind CCR7, they can induce the targeted migration of dendritic cells, and move to a dense area of T cells. On the other hand, dendritic cells chemotaxis through secretion factors CCL18, CCL19, and CCI21 can also recruit natural T cells, Dendritic cells, and activated T cells (Stein et al., 2000). Chemokines provide an effective mechanism for the mutual positioning of immune cells to ensure the smooth progress of the immune response. The migration of immune cells is closely related to the functional role it plays, and chemokine receptors provide better characteristic markers for functional lymphocyte subsets during the progressive inflammatory calcification of AS.

## Conclusion

Above all, we found that the chemokines CXCL13, CXCL8, CXCL16, CXCL5, CCL19, CCL8, and CCL18 are the most important markers of CAVD. We detected that regulatory macrophages M0, B cells memory, plasma cells, B cells naïve, NK cells activated, and macrophages M2 possibly referred to the occurrence and progress of CAVD. As the number and status of so many immune cells are significantly changed in CAVD, despite how the immune system affects AV calcification is not clear, we can infer that the immune system produces a major effect on the proceeding of calcification. In addition, these chemokines and immune cells may interact in the development of calcification in CAVD. The limitation of this study is that we did not conduct experimental studies and, thus, a deeper investigation of the immune cells can ascertain the ideal immunotherapy target and heighten autologous immunomodulatory for sufferers with CAVD.

## Data Availability Statement

The original contributions presented in the study are included in the article/Supplementary Material, further inquiries can be directed to the corresponding author/s.

## Author Contributions

DW was agreed to be accountable for all aspects of the work in ensuring that questions related to the accuracy or integrity of any part of the work are appropriately investigated and resolved and drafted the manuscript. BL and TX made substantial contributions to conception and design and revised the manuscript critically for important intellectual content. JW and KX made substantial contributions to acquisition of data. WY made substantial contributions to analysis and interpretation of data. QS gave final approval of the version to be published. All authors read and approved the final manuscript.

## Conflict of Interest

The authors declare that the research was conducted in the absence of any commercial or financial relationships that could be construed as a potential conflict of interest.

## Footnotes

1 https://www.ncbi.nlm.nih.gov/geo/query/acc.cgi?acc=GSE12644

2 https://www.ncbi.nlm.nih.gov/geo/query/acc.cgi?acc=GSE51472

3 https://david.ncifcrf.gov/

4 http://string-db.org/

5 https://cibersort.stanford.edu/

6 https://www.perl.org/

7 https://cibersort.stanford.edu/

## Supplementary Material

Supplementary Table 1 Immune Cell Infiltration of CAVD with 22 subpopulations of immune cells in 30 samples.

Supplementary Table 2 Correlation coefficients of 10 hub genes and lipid metabolism markers.

## Abbreviations

AAA: abdominal aortic aneurysm disease
AS: aortic stenosis
AVIC: aortic valve interstitial cells
BP: biological process
CAD: coronary artery disease
CAS: calcific aortic stenosis
CAT: cholesterol acyltransferase
CAVD: calcific aortic valve disease
CC: cell component
CCL: C-C Motif chemokine ligand
CCR: C-C motif chemokine receptor
CXCL: C-X -C Motif chemokine ligand
CXCR: C-X -C motif chemokine receptor
DEG: differentially expressed gene
ECM: extracellular matrix
FAS: fatty acid synthase
GO: gene ontology
HMGCR: hydroxymethylglutaryl coenzyme A reductase
KEGG: Kyoto encyclopedia of genes and genomes
LDL: low-density lipoprotein
MF: molecular function
MMP: matrix metalloproteinase
PPBP: pro-platelet basic protein
PPI: protein-protein interaction
RHD: rheumatic heart disease
TAVR: transcatheter aortic valve replacement
VCAM1: vascular cell adhesion molecule 1.
