# Supplementary material for: Predicting the Key Genes Involved in Aortic Valve Calcification Through Integrated Bioinformatics Analysis
Source: Front Genet. 2021 May 11;12:650213. doi: 10.3389/fgene.2021.650213 (PMC8144713; doi:10.3389/fgene.2021.650213)
Supplement: Supplementary Table 1 — Immune Cell Infiltration of CAVD with 22 subpopulations of immune cells in 30 samples. [file Table_1.docx]

| Immune Cell Infiltration of CAVD | | | | | | | | | | | | | | | | | | | | | | |
| --- | --- | --- | --- | --- | --- | --- | --- | --- | --- | --- | --- | --- | --- | --- | --- | --- | --- | --- | --- | --- | --- | --- |
| Tag | B cells naive | B cells memory | Plasma cells | T cells CD8 | T cells CD4 naive | T cells CD4 memory resting | T cells CD4 memory activated | T cells follicular helper | T cells regulatory (Tregs) | T cells gamma delta | NK cells resting | NK cells activated | Monocytes | Macrophages M0 | Macrophages M1 | Macrophages M2 | Dendritic cells resting | Dendritic cells activated | Mast cells resting | Mast cells activated | Eosinophils | Neutrophils |
| GSM1246205 | 0.071328261 | 0 | 0 | 0 | 0 | 0.054333455 | 0 | 0 | 0.015961833 | 0.062961003 | 0 | 0.029962792 | 0 | 0 | 0.040376579 | 0.512451588 | 0.018250862 | 0 | 0.165601207 | 0 | 0 | 0.028772421 |
| GSM1246206 | 0.112136039 | 0 | 0 | 0.097142662 | 0 | 0.012950803 | 0 | 0.015161655 | 0.062878783 | 0.021178079 | 0 | 0.019658696 | 0 | 0 | 0.082605953 | 0.456133035 | 0.024285362 | 0 | 0.074078407 | 0 | 0 | 0.021790525 |
| GSM1246207 | 0.078107163 | 0 | 0 | 0.065110392 | 0 | 0 | 0 | 0.074796307 | 0.014591057 | 0.027688899 | 0 | 0.050667548 | 0 | 0 | 0.056084256 | 0.481465816 | 0.005066608 | 0 | 0.112933889 | 0 | 0 | 0.033488067 |
| GSM1246208 | 0 | 0.011508376 | 0.017611314 | 0.112448747 | 0 | 0 | 0 | 0.05197734 | 0.037118224 | 0 | 0 | 0.042786286 | 0.002531035 | 0 | 0.14439748 | 0.443624898 | 0.062386968 | 0 | 0.057818544 | 0 | 0 | 0.015790788 |
| GSM317342 | 0.005183395 | 0.002451371 | 0.045643051 | 0.010686318 | 0.017173629 | 0.022401449 | 0 | 0.033623438 | 0 | 0.104653014 | 0 | 0.030504269 | 0.002291663 | 0 | 0.089800575 | 0.346602383 | 0.167967593 | 0 | 0 | 0.061247399 | 0.0031579 | 0.056612554 |
| GSM317343 | 0.009009139 | 0 | 0.027430881 | 0.051363488 | 0.011102755 | 0 | 0 | 0.056672123 | 0 | 0.019887972 | 0 | 0.04628836 | 0.061165777 | 0 | 0.081900189 | 0.453397399 | 0.005874628 | 0.001700337 | 0.117672153 | 0 | 0 | 0.056534798 |
| GSM317344 | 0 | 0.000928721 | 0.023398218 | 0.016900383 | 0.041883028 | 0 | 0 | 0.062287143 | 0 | 0.053061383 | 0 | 0.041044739 | 0 | 0 | 0.087696943 | 0.412877939 | 0.11252592 | 0 | 0.096048978 | 0 | 0 | 0.051346604 |
| GSM317345 | 0.016313711 | 0 | 0.027804979 | 0.012372833 | 0 | 0 | 0 | 0.055476083 | 0.004116133 | 0.054582275 | 0 | 0.04362549 | 0 | 0.043802514 | 0.05198698 | 0.490567961 | 0.056857231 | 0 | 0.110292336 | 0 | 0 | 0.032201473 |
| GSM317346 | 0.033070486 | 0 | 0.012969144 | 0.022446016 | 0.035705502 | 0.051903149 | 0 | 0.013890423 | 0 | 0 | 0 | 0.051586386 | 0.018427004 | 0 | 0.062846206 | 0.584510124 | 0.024434283 | 0 | 0.061611363 | 0 | 0 | 0.026599914 |
| GSM377368 | 0.001616197 | 0.015625724 | 0.017096348 | 0.099290568 | 0 | 0.071769383 | 0 | 0.00071603 | 0.066702658 | 0 | 0.054076289 | 0 | 0 | 0 | 0.079454824 | 0.388155082 | 0.023540715 | 0 | 0.117725041 | 0.043816265 | 0 | 0.020414875 |
| GSM377369 | 0.031701653 | 0 | 0.012060659 | 0.1539682 | 0 | 0 | 0 | 0.046310297 | 0.063876131 | 0 | 0.033129128 | 0 | 0.003162134 | 0 | 0.08887881 | 0.372650277 | 0.034863844 | 0 | 0.06847395 | 0.070950111 | 0 | 0.019974806 |
| GSM377370 | 0.025129457 | 0 | 0.015901306 | 0.053342691 | 0 | 0.021117659 | 0 | 0.035839811 | 0.078071474 | 0 | 0.050348577 | 0 | 0.013826779 | 0 | 0.092525257 | 0.457345448 | 0.075433985 | 0 | 0.032822846 | 0.037902524 | 0 | 0.010392187 |
| GSM377371 | 0.088250737 | 0 | 0 | 0.083240103 | 0 | 0 | 0 | 0.034577315 | 0.080294471 | 0 | 0.071386847 | 0 | 0.001062495 | 0 | 0.064054177 | 0.451034929 | 0.022145257 | 0 | 0 | 0.08632923 | 0 | 0.017624439 |
| GSM377372 | 0.066183688 | 0 | 0.011921379 | 0.076115793 | 0 | 0.079006135 | 0 | 0.082275894 | 0.031497865 | 0 | 0.083147271 | 0 | 0.012027999 | 0 | 0.058673163 | 0.338579886 | 0.028248246 | 0 | 0 | 0.110398638 | 0 | 0.021924044 |
| GSM1246209 | 0.03559636 | 0 | 0.016157989 | 0.03623459 | 0 | 0.059940095 | 0 | 0.059046047 | 0.045366427 | 0 | 0 | 0.055947336 | 0 | 0 | 0.049353804 | 0.517956152 | 0 | 0 | 0.085995912 | 0.013962018 | 0 | 0.024443268 |
| GSM1246210 | 0.052041503 | 0 | 0.000954322 | 0.051740246 | 0.00533001 | 0 | 0 | 0.05051594 | 0.034639725 | 0.023895492 | 0 | 0.014494001 | 0.018768133 | 0 | 0.13772353 | 0.433717812 | 0.041828356 | 0 | 0.098269467 | 0.000739429 | 0 | 0.035342033 |
| GSM1246211 | 0.068525502 | 0 | 0 | 0.015294058 | 0 | 0.068505018 | 0 | 0.006270744 | 0.039614504 | 0 | 0 | 0.034405972 | 0.00834943 | 0 | 0.069148426 | 0.460056906 | 0.001882759 | 0 | 0.218405665 | 0 | 0 | 0.009541017 |
| GSM1246212 | 0 | 0 | 0.209633639 | 0.064697247 | 0.033709667 | 0 | 0 | 0.042480614 | 0.032464995 | 0.01011644 | 0.00929234 | 0 | 0 | 0 | 0.096611285 | 0.376096106 | 0.068956511 | 0 | 0.021121134 | 0.006582849 | 0 | 0.028237174 |
| GSM1246213 | 0.005825691 | 0.002141203 | 0.014017219 | 0.001473128 | 0 | 0.119558082 | 0 | 0.009373772 | 0.013120485 | 0.030127213 | 0.004390011 | 0.003350508 | 0 | 0.343414528 | 0.051034667 | 0.344150232 | 0.013843499 | 0 | 0.024064483 | 0.012797879 | 0 | 0.007317399 |
| GSM1246214 | 0 | 0.001320192 | 0.029595347 | 0.057157299 | 0 | 0.002037016 | 0 | 0.050659347 | 0 | 0.106743254 | 0 | 0 | 0 | 0.158590256 | 0.069493432 | 0.336691768 | 0.041244194 | 0 | 0.028742179 | 0.057422815 | 0 | 0.0603029 |
| GSM1246215 | 0 | 0.014598192 | 0.035252511 | 0.041049285 | 0 | 0.042514147 | 0 | 0.054300292 | 0.022808097 | 0.023501599 | 0.027239672 | 0 | 0.034689452 | 0.036500974 | 0.10611052 | 0.390812395 | 0.029449311 | 0 | 0.082113806 | 0.033776699 | 0 | 0.025283049 |
| GSM1246216 | 0 | 0.02323895 | 0.035788233 | 0.038966112 | 0 | 0.073994556 | 0 | 0.022220463 | 0.02206455 | 0.056296761 | 0.005587162 | 0 | 0 | 0.170648131 | 0.071474681 | 0.373519767 | 0.029057273 | 0 | 0.014552105 | 0.041021988 | 0 | 0.021569268 |
| GSM1246217 | 0 | 0 | 0.04072813 | 0.05936435 | 0.008809968 | 0 | 0 | 0.055722278 | 0 | 0.065994333 | 0 | 0 | 0 | 0.090881664 | 0.101394349 | 0.395378208 | 0.025456259 | 0 | 0.037445969 | 0.042376047 | 0 | 0.076448444 |
| GSM1246218 | 0.028028394 | 0 | 0.081371521 | 0.035134118 | 0 | 0.054765584 | 0 | 0.037992119 | 0.021311441 | 0.005614138 | 0.010552799 | 0 | 0.000878033 | 0.058732132 | 0.113345902 | 0.326753788 | 0.085003286 | 0 | 0.063883554 | 0.027390429 | 0 | 0.049242762 |
| GSM317347 | 0 | 0.008702943 | 0.036596648 | 0.033749062 | 0 | 0.051986279 | 0 | 0.042131828 | 0.002072279 | 0.014125234 | 0 | 0.019101691 | 0 | 0 | 0.093693785 | 0.432682345 | 0.110228955 | 0 | 0.080198996 | 0.007771041 | 0 | 0.066958913 |
| GSM317348 | 0 | 0.004818529 | 0.012352629 | 0.012624189 | 0.057614927 | 0.018232215 | 0.050296616 | 0.004031956 | 0 | 0.068103296 | 0 | 0.010994628 | 0.016769026 | 0.002228929 | 0.11771009 | 0.399269193 | 0.079154001 | 0 | 0.102867742 | 0 | 0 | 0.042932034 |
| GSM317349 | 0.010601113 | 0 | 0.031274195 | 0.03412473 | 0.011858709 | 0.009342254 | 0.017740987 | 0.003339534 | 0.011535779 | 0.100449976 | 0 | 0.006956682 | 0.016580833 | 0.032246436 | 0.120750815 | 0.369852195 | 0.094829538 | 0 | 0.101914886 | 0 | 0 | 0.026601336 |
| GSM317350 | 0.025840372 | 0 | 0.041886949 | 0.037094616 | 0.005936672 | 0.014503095 | 0.019360089 | 0.023162549 | 0 | 0.104203781 | 0 | 0 | 0 | 0.190090382 | 0.083352999 | 0.300865405 | 0.022165987 | 0 | 0.114588957 | 0 | 0 | 0.016948147 |
| GSM317351 | 0 | 0.026131928 | 0.019354228 | 0.013992207 | 0.061907603 | 0 | 0 | 0.039647527 | 0 | 0.102422352 | 0 | 0 | 0 | 0.286701493 | 0.073559225 | 0.263883347 | 0.016782814 | 0 | 0.012399443 | 0.033553521 | 0 | 0.049664313 |
| GSM377373 | 0 | 0.054523731 | 0.028481427 | 0.081949157 | 0 | 0 | 0 | 0.022228787 | 0.049070655 | 0 | 0.041020636 | 0 | 0 | 0.169567613 | 0.089530383 | 0.359530241 | 0.022278857 | 0 | 0 | 0.064178463 | 0 | 0.01764005 |
| GSM377374 | 0 | 0.076362607 | 0.014399648 | 0.113140492 | 0 | 0 | 0 | 0.010314927 | 0.06536287 | 0 | 0.006391105 | 0 | 0 | 0 | 0.10137445 | 0.392721649 | 0.109451779 | 0 | 0.004380002 | 0.063613016 | 0 | 0.042487453 |
| GSM377375 | 0.074835868 | 0 | 0 | 0.124862204 | 0 | 0.015930482 | 0 | 0.02586636 | 0.098382365 | 0 | 0.058754995 | 0 | 0.024647147 | 0 | 0.093562014 | 0.353794265 | 0.037396385 | 0 | 0.022737982 | 0.045106528 | 0 | 0.024123403 |
| GSM377376 | 0.00951633 | 0.008057119 | 0.030277725 | 0.047267334 | 0 | 0 | 0 | 0.055239966 | 0.071851522 | 0 | 0.024196346 | 0 | 0 | 0.162738343 | 0.039791668 | 0.384786501 | 0.041106889 | 0 | 0.009633064 | 0.083141466 | 0 | 0.032395725 |
| GSM377377 | 0.017065584 | 0.023501897 | 0.013202737 | 0.1439863 | 0 | 0.009939448 | 0 | 0.043619675 | 0.050322764 | 0 | 0.041601287 | 0 | 0.002396139 | 0 | 0.080297479 | 0.483577945 | 0.007455745 | 0 | 0.065301467 | 0.002534245 | 0 | 0.015197287 |
