# Supplementary material for: Predicting the Key Genes Involved in Aortic Valve Calcification Through Integrated Bioinformatics Analysis
Source: Front Genet. 2021 May 11;12:650213. doi: 10.3389/fgene.2021.650213 (PMC8144713; doi:10.3389/fgene.2021.650213)
Supplement: Supplementary Table 2 — Correlation coefficients of 10 hub genes and lipid metabolism markers. [file Table_2.docx]

| **Correlation coefficients of 10 hub genes and lipid metabolism markers** | | | |
| --- | --- | --- | --- |
| gene1 | gene2 | cor | p value |
| CCL8 | CAT | 0.054 | 0.776352178 |
| CCL8 | CCL18 | 0.816 | 3.92E-08 |
| CCL8 | CCL19 | 0.757 | 1.32E-06 |
| CCL8 | CCL8 | 1 | 1.06E-220 |
| CCL8 | CXCL13 | 0.598 | 0.000478389 |
| CCL8 | CXCL16 | 0.866 | 6.38E-10 |
| CCL8 | CXCL5 | 0.623 | 0.000237888 |
| CCL8 | CXCL8 | 0.594 | 0.000544398 |
| CCL8 | FAS | 0.474 | 0.008068021 |
| CCL8 | HMGCR | 0.529 | 0.002648568 |
| CCL8 | LPL | 0.172 | 0.362375064 |
| CCL8 | MMP9 | 0.655 | 8.56E-05 |
| CCL8 | PPBP | 0.603 | 0.000414786 |
| CCL8 | VCAM1 | 0.856 | 1.62E-09 |
| CXCL16 | CAT | 0.079 | 0.679301609 |
| CXCL16 | CCL18 | 0.808 | 6.73E-08 |
| CXCL16 | CCL19 | 0.826 | 1.92E-08 |
| CXCL16 | CCL8 | 0.866 | 6.38E-10 |
| CXCL16 | CXCL13 | 0.601 | 0.000441731 |
| CXCL16 | CXCL16 | 1 | 0 |
| CXCL16 | CXCL5 | 0.673 | 4.62E-05 |
| CXCL16 | CXCL8 | 0.563 | 0.001206952 |
| CXCL16 | FAS | 0.532 | 0.002496527 |
| CXCL16 | HMGCR | 0.581 | 0.000757512 |
| CXCL16 | LPL | 0.148 | 0.434516357 |
| CXCL16 | MMP9 | 0.767 | 7.51E-07 |
| CXCL16 | PPBP | 0.715 | 9.12E-06 |
| CXCL16 | VCAM1 | 0.884 | 9.74E-11 |
| CXCL13 | CAT | -0.48 | 0.007291537 |
| CXCL13 | CCL18 | 0.493 | 0.005690227 |
| CXCL13 | CCL19 | 0.553 | 0.001533074 |
| CXCL13 | CCL8 | 0.598 | 0.000478389 |
| CXCL13 | CXCL13 | 1 | 0 |
| CXCL13 | CXCL16 | 0.601 | 0.000441731 |
| CXCL13 | CXCL5 | 0.803 | 9.02E-08 |
| CXCL13 | CXCL8 | 0.788 | 2.30E-07 |
| CXCL13 | FAS | 0.079 | 0.677235708 |
| CXCL13 | HMGCR | 0.025 | 0.897160358 |
| CXCL13 | LPL | -0.079 | 0.6796568 |
| CXCL13 | MMP9 | 0.734 | 3.99E-06 |
| CXCL13 | PPBP | 0.809 | 6.23E-08 |
| CXCL13 | VCAM1 | 0.48 | 0.007245008 |
| CXCL8 | CAT | -0.497 | 0.005162739 |
| CXCL8 | CCL18 | 0.428 | 0.018397239 |
| CXCL8 | CCL19 | 0.63 | 0.000189296 |
| CXCL8 | CCL8 | 0.594 | 0.000544398 |
| CXCL8 | CXCL13 | 0.788 | 2.30E-07 |
| CXCL8 | CXCL16 | 0.563 | 0.001206952 |
| CXCL8 | CXCL5 | 0.892 | 3.63E-11 |
| CXCL8 | CXCL8 | 1 | 1.06E-220 |
| CXCL8 | FAS | -0.022 | 0.90899925 |
| CXCL8 | HMGCR | -0.017 | 0.929389426 |
| CXCL8 | LPL | -0.087 | 0.648927071 |
| CXCL8 | MMP9 | 0.797 | 1.37E-07 |
| CXCL8 | PPBP | 0.798 | 1.26E-07 |
| CXCL8 | VCAM1 | 0.411 | 0.024154176 |
| CXCL5 | CAT | -0.408 | 0.025288041 |
| CXCL5 | CCL18 | 0.539 | 0.002103979 |
| CXCL5 | CCL19 | 0.697 | 1.84E-05 |
| CXCL5 | CCL8 | 0.623 | 0.000237888 |
| CXCL5 | CXCL13 | 0.803 | 9.02E-08 |
| CXCL5 | CXCL16 | 0.673 | 4.62E-05 |
| CXCL5 | CXCL5 | 1 | 0 |
| CXCL5 | CXCL8 | 0.892 | 3.63E-11 |
| CXCL5 | FAS | 0.115 | 0.546447404 |
| CXCL5 | HMGCR | 0.077 | 0.686388651 |
| CXCL5 | LPL | -0.159 | 0.401083907 |
| CXCL5 | MMP9 | 0.853 | 2.14E-09 |
| CXCL5 | PPBP | 0.91 | 3.26E-12 |
| CXCL5 | VCAM1 | 0.484 | 0.006668308 |
| CCL19 | CAT | -0.009 | 0.960901029 |
| CCL19 | CCL18 | 0.725 | 5.83E-06 |
| CCL19 | CCL19 | 1 | 0 |
| CCL19 | CCL8 | 0.757 | 1.32E-06 |
| CCL19 | CXCL13 | 0.553 | 0.001533074 |
| CCL19 | CXCL16 | 0.826 | 1.92E-08 |
| CCL19 | CXCL5 | 0.697 | 1.84E-05 |
| CCL19 | CXCL8 | 0.63 | 0.000189296 |
| CCL19 | FAS | 0.476 | 0.007895432 |
| CCL19 | HMGCR | 0.461 | 0.01039291 |
| CCL19 | LPL | 0.26 | 0.165935379 |
| CCL19 | MMP9 | 0.813 | 4.86E-08 |
| CCL19 | PPBP | 0.661 | 7.10E-05 |
| CCL19 | VCAM1 | 0.811 | 5.58E-08 |
| VCAM1 | CAT | 0.214 | 0.255293782 |
| VCAM1 | CCL18 | 0.787 | 2.44E-07 |
| VCAM1 | CCL19 | 0.811 | 5.58E-08 |
| VCAM1 | CCL8 | 0.856 | 1.62E-09 |
| VCAM1 | CXCL13 | 0.48 | 0.007245008 |
| VCAM1 | CXCL16 | 0.884 | 9.74E-11 |
| VCAM1 | CXCL5 | 0.484 | 0.006668308 |
| VCAM1 | CXCL8 | 0.411 | 0.024154176 |
| VCAM1 | FAS | 0.666 | 5.96E-05 |
| VCAM1 | HMGCR | 0.699 | 1.74E-05 |
| VCAM1 | LPL | 0.265 | 0.157047176 |
| VCAM1 | MMP9 | 0.645 | 0.000118594 |
| VCAM1 | PPBP | 0.513 | 0.003756247 |
| VCAM1 | VCAM1 | 1 | 1.73E-216 |
| CCL18 | CAT | 0.249 | 0.184427086 |
| CCL18 | CCL18 | 1 | 0 |
| CCL18 | CCL19 | 0.725 | 5.83E-06 |
| CCL18 | CCL8 | 0.816 | 3.92E-08 |
| CCL18 | CXCL13 | 0.493 | 0.005690227 |
| CCL18 | CXCL16 | 0.808 | 6.73E-08 |
| CCL18 | CXCL5 | 0.539 | 0.002103979 |
| CCL18 | CXCL8 | 0.428 | 0.018397239 |
| CCL18 | FAS | 0.59 | 0.000602637 |
| CCL18 | HMGCR | 0.579 | 0.000801874 |
| CCL18 | LPL | 0.2 | 0.289992708 |
| CCL18 | MMP9 | 0.613 | 0.00032054 |
| CCL18 | PPBP | 0.46 | 0.010594753 |
| CCL18 | VCAM1 | 0.787 | 2.44E-07 |
| PPBP | CAT | -0.348 | 0.059622175 |
| PPBP | CCL18 | 0.46 | 0.010594753 |
| PPBP | CCL19 | 0.661 | 7.10E-05 |
| PPBP | CCL8 | 0.603 | 0.000414786 |
| PPBP | CXCL13 | 0.809 | 6.23E-08 |
| PPBP | CXCL16 | 0.715 | 9.12E-06 |
| PPBP | CXCL5 | 0.91 | 3.26E-12 |
| PPBP | CXCL8 | 0.798 | 1.26E-07 |
| PPBP | FAS | 0.175 | 0.355550645 |
| PPBP | HMGCR | 0.16 | 0.398760632 |
| PPBP | LPL | -0.086 | 0.651287361 |
| PPBP | MMP9 | 0.794 | 1.60E-07 |
| PPBP | PPBP | 1 | 0 |
| PPBP | VCAM1 | 0.513 | 0.003756247 |
| MMP9 | CAT | -0.294 | 0.115178754 |
| MMP9 | CCL18 | 0.613 | 0.00032054 |
| MMP9 | CCL19 | 0.813 | 4.86E-08 |
| MMP9 | CCL8 | 0.655 | 8.56E-05 |
| MMP9 | CXCL13 | 0.734 | 3.99E-06 |
| MMP9 | CXCL16 | 0.767 | 7.51E-07 |
| MMP9 | CXCL5 | 0.853 | 2.14E-09 |
| MMP9 | CXCL8 | 0.797 | 1.37E-07 |
| MMP9 | FAS | 0.223 | 0.236209704 |
| MMP9 | HMGCR | 0.202 | 0.284134622 |
| MMP9 | LPL | 0.07 | 0.712264306 |
| MMP9 | MMP9 | 1 | 0 |
| MMP9 | PPBP | 0.794 | 1.60E-07 |
| MMP9 | VCAM1 | 0.645 | 0.000118594 |
